# Updates on the anticancer potential of garlic organosulfur compounds and their nanoformulations: Plant therapeutics in cancer management

**DOI:** 10.3389/fphar.2023.1154034

**Published:** 2023-03-20

**Authors:** Pratibha Pandey, Fahad Khan, Nawaf Alshammari, Amir Saeed, Farrukh Aqil, Mohd Saeed

**Affiliations:** ^1^ Department of Biotechnology, Noida Institute of Engineering and Technology, Noida, Uttar Pradesh, India; ^2^ Department of Biology, College of Sciences, University of Hail, Hail, Saudi Arabia; ^3^ Molecular Diagnostics and Personalized Therapeutics Unit, University of Hail, Hail, Saudi Arabia; ^4^ Department of Medical Laboratory Sciences, College of Applied Medical Sciences, University of Hail, Hail, Saudi Arabia; ^5^ Department of Medicine and Brown Cancer Center, University of Louisville, Louisville, KY, United States

**Keywords:** garlic, plant therapeutics, cancer, nanoformulations, organosulfur compounds, drug delivery, molecular mechanism

## Abstract

Garlic (*Allium sativum* L.) possesses numerous pharmacological potential, including antibacterial, antiarthritic, antithrombotic, anticancer, hypoglycemic, and hypolipidemic effects. The anti-cancer action of garlic is likely the best researched of the many advantageous pharmacological effects, and its use offers significant protection against the risk of developing cancer. A few active metabolites of garlic have been reported to be essential in the destruction of malignant cells due to their multi-targeted activities and lack of significant toxicity. The bioactive compounds in garlic having anticancer properties include diallyl trisulfide, allicin, allyl mercaptan diallyl disulfide, and diallyl sulphide. Different garlic-derived constituents and their nanoformulations have been tested for their effects against various cancers including skin, ovarian, prostate, gastric, breast, and lung, colorectal, liver, oral, and pancreatic cancer. The objective of this review is to summarize the antitumor activity and associated mechanisms of the organosulfur compounds of garlic in breast carcinoma. Breast cancer continues to have a significant impact on the total number of cancer deaths worldwide. Global measures are required to reduce its growing burden, particularly in developing nations where incidence is increasing quickly and fatality rates are still high. It has been demonstrated that garlic extract, its bioactive compounds, and their use in nanoformulations can prevent breast cancer in all of its stages, including initiation, promotion, and progression. Additionally, these bioactive compounds affect cell signaling for cell cycle arrest and survival along with lipid peroxidation, nitric oxide synthase activity, epidermal growth factor receptor, nuclear factor kappa B (NF-κB), and protein kinase C in breast carcinoma. Hence, this review deciphers the anticancer potential of garlic components and its nanoformulations against several breast cancer thereby projecting it as a potent drug candidate for efficient breast cancer management.

## 1 Introduction

Natural remedies have been used extensively to cure human illnesses since ancient times, and they are now being used as a key resource in drug development studies. The best hope for cancer patients is either early diagnosis or surgical tumor removal followed by radiation therapy and chemotherapy. Nevertheless, cancer continues to be one of the major cause of mortality across the globe and demands an urgent need for developing effective ways to combat morbidity and mortality along with high economic costs (Newman and Cragg, 2012; George et al., 2021). In-depth research on phytochemicals has shown that they have anticarcinogenic properties, which affect cancer initiation, proliferation, and progression *via* regulating numerous pathways, such as differentiation, cell proliferation, apoptosis, invasion, angiogenesis, metastasis, and migration (Basmadjian et al., 2014; Ranjan et al., 2019). *Allium sativum* L., also recognized as garlic, is a member of the Alliaceae family. The term Alliaceae comes from the Celtic word “all,” which means strong and nature’s gift to mankind. The bulbs of garlic range in color from white to pink having a pungent scent and fragrant flavor (Takagi, 2020). The therapeutic and preventive properties of garlic against numerous cancers have been assessed by several epidemiologic, preclinical, and clinical investigations. *Allium sativum* extracts have been shown to have numerous biological properties including antiviral, antiprotozoal, antibacterial, anti-inflammatory, antifungal, and antioxidant activities (El-Saber Batiha et al., 2020). The anti-cancerous potential of garlic has been validated by many preclinical studies using human cancer cells, including those of the lung, mouth, stomach, pancreas, ovary, endometrium, breast, prostate, and bone cancer (Dorant et al., 1993; Tanaka et al., 2006). Further, laboratory experimentation has demonstrated that chemical components reported in garlic can repair DNA damage, induce cancer cell growth arrest, and reduce inflammatory responses.

Nanotechnology aims to improve the ability of drugs to effectively treat cancer by ensuring that they are delivered to the right target at the right times. Recently, nanotechnology-based delivery approaches have drawn wider attention as a potential solution to issues related with drug solubility and bioavailability, toxicity, and distribution. Novel approaches incorporating the therapeutic characteristics of garlic extracts and their constituents, either alone or in nanoparticle formulations, may thus result in more efficient and effective anticancer activity (Charron et al., 2015; Mandal et al., 2019; Zhang et al., 2020). Nanobiotechnology has gained attention in the last 20 years as a field of study that has influenced the development of new medications. Due to their low volume/surface ratio and several advanced and novel physiochemical properties, such as color, solubility, strength, toxicity, magnetic, optical, and thermodynamics, nanomaterial, the topic of our investigation, differ significantly from their macro scale counterparts in terms of capabilities. Mechanism associated with nanoparticles biogenesis include reduction of metallic ions of numerous biomolecules found in living things. The manufacture of vast amounts of well-defined, contaminant-free nanoparticles with defined size and morphology is made possible by nanoparticle biogenesis, which also lessens the environmental impact of biological synthesis. Recent nanotechnological advancements demonstrated the synthesis of nanoparticles utilizing the potential of microorganisms and plant based materials (tissue and extracts) including garlic constituents.

Garlic and its derived components potentially reduced the progression of tumor in animal model and suppressed cancer cell growth. In conventional medicine, *A. sativum* L. is used to prevent, and treat a number of malignancies, including cancers of blood, breast, prostate, ovarian, and gastrointestine. Major active phytoconstituents of *A. sativum* include diallyl thiosulfinate, methyl allyl trisulfide, diallyl tetrasulfide, S-allyl cysteine sulfoxide, dipropyl sulfide, dially trisulfide, 3-vinyl-4H-1,2-dithiin, allyl methane sulfinate, diallyl disulfide, dipropyl disulfide, allyl methyl thiosulfinate, S-methyl cysteinesulfoxide, 3-vinyl-6H-1,2-dithiin, dimethyl disulfide, dimethyl sulfide, methylpropyl disulfide, S-propyl cysteine sulfoxide, 3-vinyl-6H-1,3-dithiin, and, methyl methane sulfinate. Garlic derivatives including diallyl disulfide, are found to be highly effective in suppressing the growth of breast cancer cells. Mechanisms behind this growth suppression include the activation of metabolizing enzymes for carcinogen detoxification, inhibition of reactive oxygen species (ROS) generation and DNA adduct formation, control of the cell cycle, and apoptosis induction. Thus, we have focused our review on deciphering the anticancer potential of garlic against breast cancer in order to elucidate the potent drug candidate for effective management of breast cancer.

Breast cancer is one of the most common neoplasm in women worldwide, in both industrialized and developing nations. The invasion and proliferation of breast cancer have been lessened by using available therapies such as chemotherapy, hormone and radiation therapy, and/or surgery. Scientists are looking for more effective treatments for breast cancer, such as natural chemicals, as the majority of chemotherapeutic medications are linked to drug resistance, cancer relapse, and adverse effects. Apoptosis is induced and metastasis is inhibited by specific natural molecules, substances originating from living things, which inhibit cancer progression. These bioactive molecules have displayed significant efficacy in advanced stage of several cancers including breast cancer with increased survival rate and reduced morbidity (Noel et al., 2020; Sung et al., 2021). Here, we also review organosulfur compounds that have been shown in numerous studies to have anti-cancer properties against breast cancer cells. These organosulfur compounds encourage cell death, prevent breast cancer formation, and slow the growth of cancerous cells. There are very limited reviews discussing the potential anticancer efficacy of new formulations of garlic extracts and their constituents in breast cancer. In-depth descriptions of how garlic, its bioactive components, and its nanoformulations work against various malignancies are provided in this review, which also considers the potential for turning these substances into medications that fight cancer.

## 2 Major active constituents of *Allium sativum* L

The anticancer potential of numerous sulfur-containing compounds and other bioactive constituents of garlic has been extensively studied. Garlic has high concentrations of zinc, potassium, sulfur, phosphorus, and a moderate level of selenium, magnesium, calcium, iron, sodium, manganese, and vitamins (A, B and C). In recent years numerous bioactive compounds have been identified in garlic including polyphenols, flavanols, flavonoids, saponins, tannins, sulfur-containing compounds (such as alliin, ajoene, allicin, DATS, allylpropyl disulfide, S-allylcysteine (SAC), vinyldithiins, poly-saccharides, enzymes (including myrosinase, allinase, and peroxidase) and other com-pounds including phellandrene, *ß*-phellandrene, linalool, geraniol, and citral (Ansary et al., 2020). Table 1 summarizes the best reported phytoconstituents of garlic that have displayed significant anticancer potential. Allicin is the main sulfur containing active compound, and it produces a large number of allyl sulfur compounds that are oil-soluble and have anticancer properties (Bat-Chen et al., 2010; Chen et al., 2016; Zou et al., 2016; Dhanarasu, 2017). Two different non-volatile organosulfur compounds, L-cysteine sulfoxides and γ-glutamyl-L-cysteine peptides (having γ-glutamyl-S-allyl-L-cysteine), are present in whole, intact garlic cloves. When raw garlic cloves are crushed, mashed, or chewed, an enzyme called alliinase is created. The enzyme alliinase breaks down the amino acid allicin into 2-propenesulfenic acid, releasing pyruvic acid and ammonia in the process. At room temperature, 2-propenesulfenic acid is highly reactive and unstable. Two molecules of 2-propenesulfenic acid spontaneously interact to form allicin and eliminate water (Shukla and Kalra, 2007; Omar and Al-Wabel, 2010; Yun et al., 2014). Diallyl trisulfide (DATS), diallyl disulfide (DADS), allyl methyl sulphide (AMS), and diallyl sulphide (DAS) are some of the organosulfur compounds that are produced when allicin breaks down and are soluble in fat (Shang et al., 2019). It additionally yields ajoene when fermented with organic solvents. To create S-allyl mercapto cysteine (SAMC), allicin can interact with L-cysteine in the body. It was discovered that the skin of garlic contained FO (N-trans-feruloyloctopamine), a hydroxycinnamic acid derivative (Viswanathan et al., 2014; Wu et al., 2015). Natural organoselenium compounds named MSeC (Se-methyl-L-selenocysteine), a derivative of S-methyl cysteine, have been reported in garlic. The dipeptide glutamyl-S-allyl-L-cysteine, which is water soluble, is reported as peptide-glutamyl-L-cysteine. During formation of aged garlic extracts, SAC and SAMC (water-soluble organosulfur compounds) are produced from glutamyl-S-allyl-L-cysteine after prolonged fermentation of crushed garlic in an aqueous solution (Block et al., 1996; Block, 1997). From aged garlic extract, S-benzyl-cysteine (SBC), a structural analog of SAC that is water soluble, was isolated and studied. S-propargyl-cysteine (SPRC) is a derivative of SAC, an H_2_S donor has been prepared from garlic extract (Rose et al., 2005; Rose et al., 2019).

**TABLE 1:** Phytoconstituents of garlic (Data retrieved from SWISS ADME software).

Major active constituents	Full form	Chemical structure	Pubchem ID	GI absorption	BBB permeant
γ-glutamyl-S-allyl-L-cysteine	gamma-L-glutamyl-[(S)-allyl]-L-cysteine	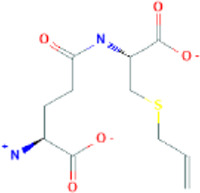	91,820,320	Low	No
Alliin	S-allyl-L-cysteine sulfoxide	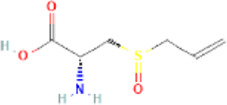	9,576,089	High	No
Allicin	S-allyl prop-2-ene-1-sulfinothioate	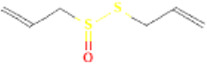	65,036	High	Yes
DATS	Diallyl trisulfide	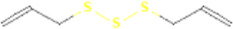	16,315	High	Yes
DADS	Diallyl disulfide	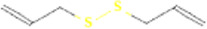	16,590	High	Yes
DAS	Diallyl sulfide	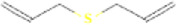	11,617	High	Yes
AMS	Allyl methyl sulfide	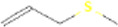	66,282	High	Yes
Thiacremonone	2,4-Dihydroxy-2,5-dimethylthiophen-3(2H)-one	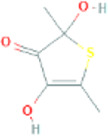	539,170	High	No
Ajoene	(E)-Ajoene	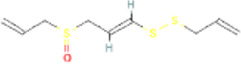	5386591	High	No
SAMC	S-allyl mercapto cysteine	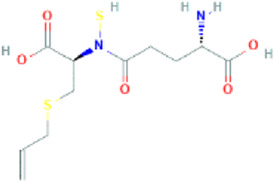	129847556	Low	No
FO	N-trans-feruloyloctopamine	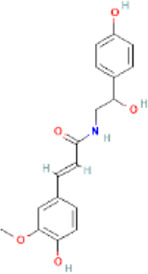	24096391	High	No
MSeC	Se-methyl-L-selenocysteine	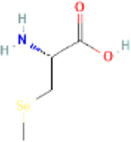	147,004	High	No
SAC	S-allyl-L-cysteine	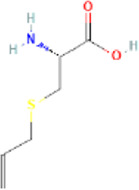	9793905	High	No
SBC	S-benzyl-cysteine	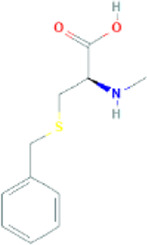	13817365	High	No
SPRC	S-propargyl-cysteine	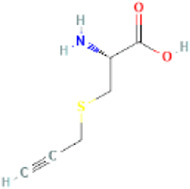	22789047	High	No

## 3 Anti-cancer efficacy of garlic extracts and their nanoformulations in breast carcinoma

The most common neoplasm in women worldwide, in both industrialized and developing nations, is breast cancer. Over 2 million new cases of female breast cancer are anticipated, along with 684,996 cancer-related fatalities worldwide in 2020. Among women, it is the primary cause of cancer and the most frequently diagnosed type. The invasion and proliferation of breast cancer have been lessened by using available therapeutics such as chemotherapy, hormone therapy, radiation therapy, and surgery. Lipodox™, Doxil™ (pegylated liposomal doxorubicin), and Abraxane™ (nanoparticulate albumin-based paclitaxel therapeutic) have been clinically utilized as a potent component of current combinatorial chemotherapies against triple negative breast cancer over conventional drug therapies. Myocet liposomal™ (non-pegylated liposome) has been approved for its usage in triple negative breast cancer along with vinorelbine or cyclophosphamide. Taxol™ (carrier free paclitaxel solution) with Cremophor EL has also got approval for its usage triple negative breast cancer (Ghanbari-Movahed et al., 2021; Ghosh et al., 2021; Miguel et al., 2022). However, these therapies still associated with life threatening toxicities.

Garlic is recognized to have potent anticancer efficacy due to the presence of several organic and sulfur components. These compounds affect multiple stages of cancer cell proliferation, development, growth, migration, invasion, and metastasis by interfering with cell cycle regulation, inhibiting cell signaling pathways, apoptosis induction, autophagy, and antioxidant potential (Shang et al., 2019; Zhang et al., 2020; De Greef et al., 2021). The sulfur compounds in garlic are both fat- and water-soluble and demonstrate anticancer effects by reducing oxidative stress, metabolizing carcinogens, and boosting immune response (Miroddi et al., 2011; Lan et al., 2013). The therapeutic potential of garlic nanoformulations was higher than that of the photo component alone. For instance, allicin nanoparticles showed a stronger anti-angiogenesis impact than allicin by itself (Shafeeque and Hashim, 2018). In addition to providing targeted drug delivery, the use of NDDSs for drug delivery can in-crease the bioavailability of medications that are poorly water-soluble, allow for the co-delivery of several medications, prevent drug toxicity in normal cells, and extend the duration of therapeutic activity. In order to show their potential anticancer actions, nanoformulations have been used to deliver parent chemicals directly to the crucial locations of the malignant tissues. Nanoscale materials are used as carriers for delivering medications to their sites of action in NDDSs, which is a rapidly evolving field of science. ROS and cytotoxicity investigation revealed enhanced antitumor potential of DADS when incorporated with solid lipid nanoparticles in comparison to DADS-free drug in breast cancer cells (Nkrumah-Elie et al., 2012). The anticancer potential of key constituents of garlic extracts, and their nanoformulations should therefore be thoroughly studied. The growth of MCF-7 human breast cancer cells was inhibited by an aqueous preparation of allicin and garlic powder. Growth arrest of cancer cells in G2/M or G0/G1 phase of cell cycle were reported in conjunction with growth inhibition. The growth-inhibitory impact of allicin was closely related to the reduction in intracellular glutathione (GSH) levels in both MCF-7 (luminal-A) and HCC-70 (triple-negative) breast cancer cells (Hirsch et al., 2000; Chen et al., 2016; Rosas-González et al., 2020). While both alliin and allicin reduced cell viability of MCF-7 (luminal-A) and HCC-70 (triple-negative) breast cancer cells, allicin showed more pronounced cytotoxicity and antiproliferative activity in both cells by lowering mitochondrial membrane potential, apoptotic induction, and enhanced expression level of various caspases. Additionally, it increased the expression level of proapoptotic proteins (p21, Noxa, and Bak) while downregulated the expression level of Bcl-xL antiapoptotic protein (Tsubura et al., 2011; Milczarek, 2020; Fatima et al., 2021; Catanzaro et al., 2022). In contrast to allicin, alliin caused senescence in both MCF-7 (luminal-A) and HCC-70 (triple-negative) breast cancer cells in a dose dependent manner (Farhat et al., 2021).

Oil-soluble components from garlic, namely, DAS, DADS, and DATS, significantly reduced the proliferation of CMT-13 (canine mammary tumor) cells in comparison to water soluble organosulfur compounds. While DATS was cytotoxic, DAS and DADS were cytostatic. The cancer-suppressing potency was enhanced with an increase in intracellular GSH levels in addition to the number of sulfur atoms (Sundaram and Milner, 1993; Nakagawa et al., 2001; Lei et al., 2008). DADS also showed cytotoxicity toward the MCF-7 (ER-positive human breast cancer) cells, T47D (differentiated epithelial sub strain), KPL-1 (created from the malignant effusion of a breast cancer patient), MDA-MB-231 and MKL-F (ER-negative) human breast cancer cells (Altonsy et al., 2012; Na et al., 2012; Hahm et al., 2021). In MDA-MB-231 cells, it resulted in cell cycle arrest (sub-G1 phase) and apoptotic induction. This was accompanied by modulations in the expression levels of numerous proteins such as Bax, Bcl-xL, and caspase-3.

DADS presented significant cytotoxicity against MCF-7 cancer cells *via* apoptosis induction, increased PARP and caspase-3 cleavages, activated SAPK/JNK/p38 pathways and suppressed extracellular ERK/MAPK cell signaling pathways (Lei et al., 2008; Jun et al., 2009). Diallyl trisulfide (DATS) treatment significantly increased growth arrest at the G2/M phase in both MCF-7 and MDA-MB-231cells when compared to control cells, which is consistent with the RNA seq findings. In addition, DATS administration dramatically elevated the Ser10 phosphorylation of histone H3 (mitotic marker) in both breast cancer cells. However, DADS did not affect SLIT/ROBO signaling in breast cancer cells (Huang et al., 2015). Annexin V/propidium iodide (PI) staining in DATS-treated MCF-7 cells indicated apoptotic cell death with an increased growth arrest at G0/G1 phase. In MCF-7 cells, DATS triggered the Bcl-2 (anti-apoptotic protein) phosphorylation and the proteolytic cleavage of PARP (poly ADP-ribose polymerase). MCF-7 cells treated with SP600125 (JNK inhibitor) or transiently transfected with dominant-negative JNK experienced a reduction in DATS-induced apoptosis and increased ROS generation. N-acetyl-l-cysteine prevented DATS-induced apoptosis and JNK activation. Additionally, DATS-treated MCF-7 cells demonstrated enhanced AP-1 DNA binding activity, which was suppressed by NAC and the JNK inhibitor. The cells treated with c-jun siRNA were resistant to proteolytic PARP cleavage in DATS treated cancer cells. Female Balb/c mice given 5 mol/kg DATS orally were unable to generate human MCF-7 cell tumor xenografts (Nkrumah-Elie et al., 2012). Altonsy et al. (Altonsy et al., 2012) corroborated these findings and reported that DADS inhibited the growth of MCF-7 carcinoma cells *via* cell cycle arrest at sub-G0 phase and apoptotic induction. Additionally, DADS promoted H4 histone hyperacetylation by inducing phosphatidylserine translocation to modulate Bax, Bcl-xL, Bcl-2, and Bcl-W protein expression levels and caspase-3 activation. Another study reported DADS cytotoxicity toward MCF-7 cells with reduced cell proliferation and apoptosis induction *via* intrinsic signaling pathways associated with upregulated protein (Bax, Bad, and caspases) expression levels and downregulated Bcl-2 protein expression levels (Xiao et al., 2014). DADS treatment further resulted in reduced cell growth of several human triple-negative breast cancer cells (MDA-MB-231, BT-549, and MDA-MB-468) *via* regulating *ß*-catenin signaling pathway. Increased expression of Bax, caspase-3, and caspase-9, as well as decreased levels of Bcl-2, matrix metallo-proteinase-9 (MMP-9), and *ß*-catenin siRNA, were associated with the induction of apoptosis and inhibition of cell proliferation (Malki et al., 2009; Nkrumah-Elie et al., 2012; Li et al., 2018). MDA-MB-231 cells treated with DADS had higher levels of miR-34a expression. The SRC/Ras/ERK signaling pathway was finally blocked by further suppression of SRC expression (Cao et al., 2014). In MCF-10A cells (typical human mammary epithelial cells), DATS reduced the ability of benzo [a]pyrene to cause cancer by preventing cell division, causing cell cycle arrest at the G2/M phase, and inhibiting the production of ROS and DNA damage (Kiesel and Stan, 2017).

DATS treatment induced cell cycle arrest (G2/M phase) and apoptosis, which drastically reduced MCF-7 cell viability and not affected the MCF-12A normal human breast epithelial cells. Enhanced translocation of p53 from the cytoplasm into the nucleus was associated with the activation of apoptosis, along-with upregulation of the Bax and p53 protein expression levels. Akt and Bcl-2 protein expression levels were further inhibited, whereas FAS and cyclin D1 protein expression levels were elevated (Liu et al., 2017). Cancer stem cells (CSCs) from the MCF-7 and SUM159 cell lines of two human breast cancers were used to create tumor spheres, however, DATS blocked this by lowering the levels of numerous proteins CD44, ALDH1A1, Nanog, and Oct4. Apoptosis was found to inhibit cell proliferation *via* modulating Bax, caspases, cyclin D1, Bcl-2, caspases and proliferating cell nuclear antigen (PCNA) expression levels. Further it has been shown that DATS inhibited the aberrant cell signaling pathway associated with carcinogenesis such as Wnt/β-catenin (regulator of breast CSCs). Exposure of DATS in breast cancer cells has enhanced c-Myc and GSK-3β expression levels while downregulated the expression levels of phospho-glycogen synthase kinase-3β (p-GSK-3β), and *ß*-catenin (Johnston, 2010).

Kim and colleagues noted downregulation of numerous cell components including that Forkhead transcription factor Forkhead box Q1 (FoxQ1) protein, Dachshund homolog 1 (DACH1) and aldehyde dehydrogenase 1 (ALDH1) in DATS-mediated CSC suppression. Trx-1 expression and Trx-1 reductase enzyme activity were decreased by DATS in breast cancer cells (MDA-MB-231, ZR-75–1, Hs578T, and MCF-7) (Malla et al., 2021). The cysteine residues in thioredoxin-1 (redox-active disulfide/dithiol) containing 12kD protein, are converted to an oxidized disulfide bond (intra-molecular Trx-1-S2) configuration through a chemical process. Trx-1, which is released by tumor cells, speeds up the growth of tumors and reduces patient survival. Trx1 overexpression in tumor cells caused the JNK/p38 signaling pathway-associated apoptosis signal-regulating kinase 1 (ASK1) expression to be suppressed, which in turn suppressed apoptosis. Another study revealed DATS mediated growth inhibition of MDA-MB-231, MCF10A-H-Ras, and MCF-7 breast cancer cells by reducing secretases expression levels such as ADAM-17 and ADAM-10 (a disintegrin and metalloprotease-10), that are linked to the activated Notch signaling pathway. Additionally, it inhibited Jagged-2 and Jagged-1 (Notch ligands) associated with activated Notch signaling pathway (Kim et al., 2016).

DATS reduced transforming growth factor-β1 (TGF-β1) and thromboxane B2 (TXB2) production *via* blocking platelet activation and aggregation (by platelet-activating factor), and hence resulted in inhibition of platelet-mediated hematogenous metastasis of MDA-MB-231 cells (Hahm and Singh, 2014; Liu et al., 2015; Li et al., 2020). DATS suppressed ER-protein levels, a novel target of the drug in two potent breast cancer cells, MCF-7 and T47D. Along with reduced nuclear ER-protein levels and inhibition of ER-mRNA, the expression levels of cyclin D1 and pS2 were also downregulated. DATS treatment also resulted in downregulation of overexpressed Peptidyl-prolyl cis-trans isomerase (Pin1), which further restrained the activity of ERE2e1b-luciferase reporter (Sigounas et al., 1997). By suppressing mRNA expression levels of Bcl-2, cyclin D1, Bcl-xL, MMP-2, and vascular endothelial growth factor (VEGF) in MDA-MB-231 and MCF-7 breast cancer cells, DATS intervention significantly reduced leptin-induced cell proliferation, clonogenic cell viability, invasion and migration potential. DATS also displayed a strong antimetastatic effect by reducing MMP-2/9 expression levels in triple-negative breast cancer cells (HS 578t and MDA-MB-231 cells) and ultimately blocked both the NF-κB and ERK/MAPK signaling pathway (Ip et al., 1992; Zhang et al., 2014). In MCF-7 cells, DATS treatment induced ROS mediated apoptosis and subsequent activation of AP-1 and JNK, together with enhanced ROS accumulation (Cao et al., 2014).

Another *in vivo* study reported reduced STAT3 expression levels in DATS treated breast cancer xenografts in mice. Further, DATS treatment decreased the incidences of breast cancers in xenografted MDA-MB-231 tumor models *via* reducing MMP-2 and -9 expression levels (Wei et al., 2017; Liu et al., 2018; Vijayakumar et al., 2019). DATS (natural histone deacetylase inhibitor) treatment prevented the MDA-MB-231 hypoxia-inducing cell metastasis in embryonic zebrafish tumor model *via* blocking the HIF-1 transcriptional activity. Furthermore, it reduced EMT-related proteins (Slug, MMP-2 and Snail), VEGF-A and L1CAM protein expression levels. In female ACI rats (an inbred line derived from a cross between Copenhagen and August strains), DAS reduced the concentrations of diethylstilbestrol (DES)-induced lipid hydroperoxides (Selvan et al., 2018).

SAMC, a stable organosulfur derivative from garlic, displayed potent anti-proliferative properties against MCF-7 breast cancer cells (Sigounas et al., 1997). MCF-7 and MDA-MB-231 cells displayed potent growth inhibition after SAMC treatment due to increased p21 and p53 expression levels, induction of apoptosis, cell cycle arrest (G0/G1 phase), increased caspase-9, and caspase-3 expression levels in relation to the mitochondrial apoptotic pathway, reduced Bcl-xL and Bcl-2 expression levels. SAC dramatically slowed the development of MDA-MB-231 breast carcinoma cells in a dose and time dependent manner. Increased E-cadherin expression and decreased MMP-2 expression levels in MDA-MB-231 cells after SAC therapy imply that cell proliferation, adhesion, and invasion are inhibited, which helps to prevent metastasis.

Using a rat model of DMBA-induced mammary tumors, it is shown that the use of garlic powder successfully inhibited the growth of tumors and validated the fact that DADS has been more active than AMS and DAS. According to Schaffer et al., supplementation with garlic powder, SAC, and DADS reduced the incidence of mammary cancer in rats, hence preventing the development of mammary carcinogenesis brought on by N-methyl-N-nitrosourea (MNU) (Schaffer et al., 1996; Petrovic et al., 2018; Xiong et al., 2018). 67NR breast cancer cell, which was implanted in mammary pads of BALB/c mice, demonstrated anticancer activity by being inhibited by garlic extract. Additionally, by reducing the DMBA-DNA interaction to lower the incidence of mammary tumors, in the long run, garlic powder prevented DMBA-induced mammary cancer tumors in rats. Among the numerous organosulfur compounds, garlic compounds that are oil-soluble have been shown in rodents to have stronger anticancer action than those that are water-soluble. DMBA, 2-amino-1-methyl-6-phenylimidazo [4,5-b] pyridine (PhIP) and MNU induced mammary cancer in rats were significantly inhibited by DADS (Zhang et al., 2014). After subcutaneous injection of MCF-7 and MDA-MB-231 cells in nude mice xenograft tumor models were created that showed anticancer and antiproliferative behavior from DADS. DADS treatment lead to the activation of tristetraprolin expression and significant downregulation of MMP-9 protein and urokinase-type plasminogen activator expression (Nakagawa et al., 2001). DADS demonstrated reduced growth proliferation by reducing initial tumor weight in female nude mice (with orthotopic transplantation, KPL-1), resulting in growth inhibition in human breast cancer cells (*in vitro* and *in vivo*) (Yi and Su, 2013; Kim et al., 2020). Table 2 summarizes the mechanism of garlic and its constituents by which they slow anticancer activity against breast cancer cells.

**TABLE 2:** Anticancer effects of garlic constituents on breast cancer.

Components of garlic	Mechanism of action	References
DATS	• DATS treatment induced apoptosis in MCF-7 cells with Bax (pro-apoptotic protein) and p53 induction and offered a potent strategy for breast cancer management	Hahm and Singh (2014)
	• Apoptotic induction *via* reducing peptidyl-prolyl cis–trans isomerase (Pin1) protein expression level in T47D and MCF-7 cells	Liu et al. (2015)
	• Inhibited metastasis and MMP2/9 activity of TNBC cells *via* down-regulating transcriptional activities of ERK/MAPK and NF-κB signaling pathways	Kim et al. (2016)
	• Inhibition of leptin induced oncogenic signaling in MCF-7 and MDA-MB-231 *via* reducing STAT3 protein expression	Liu et al. (2018)
	• Inhibited the Trx-1 expression and enzymatic activity of Trx reductase in breast cancer cells	Wei et al. (2017)
	• Inhibited HIF-1α transcriptional activity and hypoxia-induced hematogenous metastasis in a dose-dependent manner in MDA-MB-231 cells. Induced ROS production and apoptotic cell death in MCF-7 and MDA-MB-231 breast cancer cells	Chandra-Kuntal et al. (2013)
SAMC (aged garlic)	Displayed growth inhibitory potential in MCF-7 breast cancer cells	Sigounas et al. (1997)
DADS	DADS alienated the efficacy of linoleic acid (breast cancer cell stimulator) and synergized the efficacy of eicosapentaenoic acid (breast cancer cell suppressor) in MDA-MB-231 cells thereby projecting it as a strong modulator of breast cancer cell proliferation	Nakagawa et al. (2001)
Diallyl disulfide	Prevented mammary cancer growth in female rats	Ip et al. (1992)
Ajoene	Induced growth arrest and apoptosis *via* targeting protein folding in the endoplasmic reticulum of in MDA-MB-231 cancer cells	Kaschula et al. (2016)

**FIGURE 1:**
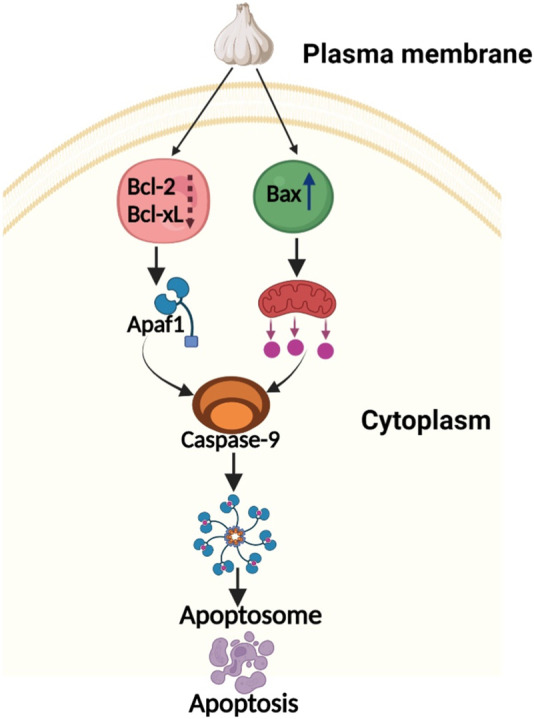
Proposed pathway for garlic induced apoptosis in breast cancer.

Despite strong anticancer activity of various garlic derived plant therapeutics, these compounds suffer from oral bioavailability and off target toxicity. Therefore, several nanoformulations have been tried to improve the bioavailability of these compounds. Garlic extracts contain anticancer chemicals can combine to generate nano-conjugates that can help stop the spread of cancer cells. When administered at a concentration of 100 g/mL, garlic clove extract-mediated silver nanoparticles (G-AgNPs) displayed cytotoxic action against the MCF-7 cell line. Additionally, it affected the nucleus morphology of MCF-7 cells, causing cell clumping and membrane instability (Menon et al., 2012). G-AgNPs did not cause any toxicity or mortality in neonates of *Corylus cornuta*. In a different study, garlic extract-mediated silver nanoparticles (Ag-S2) caused cytotoxicity in the MCF-7 cancer cell line by reducing cell viability in a concentration-dependent manner (Ahamed et al., 2022). Nevertheless, MCF-7 cells were not toxicated by the gold nanoparticles (G-AuNPs) produced by garlic extract. Superparamagnetic hematite nanoparticles made from garlic extract were synthesized, and their cytotoxicity against the breast cancer MCF-7 cell line was examined. The findings imply that at an IC_50_ of 346.25 mg/mL, the cell proliferation was inhibited in a dose responsive manner (Talluri et al., 2017). Even ZnO-reduced graphene oxide nanocomposites (ZnO-RGO NCs) made from the extract of garlic cloves showed increased cytotoxicity against MCF-7 cells (Kim et al., 2020). DADS nanoparticles (solid lipid) demonstrated higher cytotoxicity than DADS alone against MCF-7 carcinoma cells by inducing apoptosis (*via* intrinsic signaling pathway) associated with elevated expression levels of Bax, Bad, caspase-3, and caspase-9, and decreased protein expression level of Bcl-2 (Lee et al., 2015). Figure 1 presents a proposed pathway for garlic induced apoptosis in breast cancer.

## 4 Clinical trials of garlic products and constituents in breast cancer

Five grams of crushed garlic (raw) enhanced the expression of several genes, including the proto-oncogene c-Jun, the aryl hydrocarbon receptor, nuclear translocator, HIF-1A (hypoxia-inducible factor 1A), oncostatin M (OSM), and the viral oncogene homolog (V-rel avian reticuloendotheliosis) in a clinical investigation involving 17 volunteers. These findings indicated that garlic components are multifaceted and comprise activation of genes associated with immunity, xenobiotic metabolism, and apoptosis in humans and Mono Mac six cells. (Charron et al., 2015). Similar findings were made in clinical research that involved 20 cancer patients and found that raw garlic and allicin had cytotoxic effects against a variety of cancer types (Bespalov et al., 2004). Garlic supplements were reported beneficial in the management of breast cancer (benign) patients, where they resulted in the suppression of breast fibromatosis. Furthermore, garlic intake also reduced docetaxel removal in patients having CYP3A5*1A allele (Zuniga et al., 2019). Garlic consumption can be increased to promote health, according to a randomized controlled trial of breast cancer survivors (Ishikawa et al., 2006). A dose of four capsules per day for 12 weeks of aged garlic extract (AGE) treatment significantly enhanced number of natural killer (NK) cells and its activity in patients with advanced lung, colon, pancreatic and liver cancer. Each capsule included 500 mg of AGE, 11 mg of sucrose fatty acid ester and 727 mg of crystalline cellulose. Respective doses were ceased after 12 weeks, with a follow-up after further 12 weeks to monitor the number of NK cells and its activity (Tanaka et al., 2006). Additionally, aged garlic extract inhibited cell growth and minimized the occurrence of human colorectal adenoma *via* modulating numerous cell signaling path-ways (Millen et al., 2007). Raw garlic consumption (five pyramid doses per day) for a duration of 12 months, with a follow-up after 12 months, was found to lessen the risk of colorectal adenoma in a randomized controlled trial on 57,560 patients (Ma et al., 2012).

Long-term garlic supplementation was further associated with reduced risk of mortality in patients with stomach cancer. For a period of 7 years, a dosage of 200 mg of aged garlic extract and 1 mg of steam-distilled garlic oil was administered twice daily. In this randomized intervention study, 3,365 participants were used to investigate the incidence and mortality of stomach cancer using a 2 2 factorial design. Follow-up period is initially 7.3 years, and it is later extended to 22.3 years (Li et al., 2019; Guo et al., 2020). Garlic supplementation had more pronounced protective impact on gastric cancer prevention when combined with abstinence from alcohol. This randomized clinical trial found a relationship between smoking and an increased risk of gastric cancer incidence and death. After a follow-up period of 22.3 years, more pronounced anti cancerous effect was reported after garlic supplementation in gastric cancer when the individuals abstained from alcohol (You et al., 2001). Garlic formulations slowed the evolution of pre-cancerous gastric lesion, and examinations of serum samples further demonstrated that participants in the participating treatment groups had significantly greater S-allyl cysteine (SAC) doses (Zhang et al., 2008). Allicin was injected into the lesion site of individuals with gastric cancer *via* a gastroscopy in a different study that involved a randomized controlled trial with 80 participants. The medication was given to the patient 48 h before surgery.

Laboratory testing of the gastric cancer tissue removed after gastrectomy demonstrated that allicin had the ability to impede cancer cell proliferation *via* apoptotic induction, cell cycle arrest (at G0/G1 phase) and modulated Bax and Fas (upregulated), Bcl-2 (downregulated) expression levels (Gatt et al., 2015). Allin and allicin, two garlic constituents, have been shown to protect patients against the side effects of chemotherapy (including febrile neutropenia). This was frequently seen in chemotherapy patients with hematological malignancies. Patients who participated in a 101-patient randomized controlled experiment received two doses of 450 mg of allicin and allin daily. The patients were monitored daily until the neutropenia was resolved by incessant monitoring of complete blood count (CBC) (Tilli et al., 2003). In another clinical trial, aqueous garlic extract showed preventive properties against prostate carcinoma by increasing flow and frequency of urine. Despite the fact that the follow-up period was unreported, the trial was carried out on 27 patients (daily dose of 1 mL/kg body weight) for 30 days. A study using 21 patients found that the active ingredient in garlic, ajoene, had anticancer properties against skin cancer. The tumor size was reduced after topical application of 0.4% ajoene cream (400 mg ajoene, 0.3 ml sorbitonoleate and 0.3 ml polysorbate 80 in 100 ml 1% of carbomeric gel) as shown by activated apoptotic pathways and modulated Bcl-2 expression levels (Shashikant et al., 1985).

Evaluation of dietary supplements and medications used for health purposes requires documentation of their efficacy and safety. The ways of processing garlic can influence its effectiveness and safety. However, there is no recommended everyday garlic in-take, the German Commission E monograph from 1988 advised that a per day intake of 12 cloves (or about 4 g) of garlic is good for human health. Fresh garlic, garlic oil, garlic powder, and AGE are the four main categories into which commercially produced garlic preparations fall. Even though garlic has been reported safe when used as a complementary (or condiment) agent, safety concerns still need to be addressed. There are three things to keep in mind when using garlic: a) allicin remains one of the key irritant in raw garlic; b) oil-soluble OSCs reported to be more toxic in comparison to water-soluble OSCs and c) toxicity associated with OSCs becomes much less harmful over time. When allicin was administered directly into the intestinal tract of rats, the intestinal linings were harmed and the gut flora was altered using enteric-coated garlic products. The mucosal epithelial membrane may become damaged by fresh garlic (Amagase et al., 2001; Macan et al., 2006). A number of studies, including those for teratogenicity, mutagenicity, acute and subacute toxicity, and chronic toxicity, have shown that AGE is relatively safe. Recent clinical trials demonstrated that, when used as a supplemental drug, AGE was safe for patients receiving warfarin therapy (Song and Milner, 2001). Because there are so many different garlic preparations available, the medicinal consequences are also diverse, suggesting that some preparations could have negative effects. A monograph that outlines the process and requirements for AGE has been created by the U.S. Pharmacopoeia and other organizations. Garlic preparations should be consumed while considering their potential side effects, metabolism, drug-synergistic interactions, interference with vital enzymes, and impact on normal microflora. Consumers could make better decisions if labels included recommendations based on laboratory and clinical findings. Additionally, the anticancer properties of uncrushed garlic will be reduced when heated in the microwave or oven (Nakagawa et al., 2001; Mondal et al., 2022). Garlic has been shown to have anticancer properties by obstructing various phases of carcinogenesis. The nutritional or chemopreventive benefits of garlic, however, go far beyond the idea that it has therapeutic effects against cancer. To investigate the novel properties of garlic, more carefully planned experiments and trials are needed. (Hirsch et al., 2000).

## 5 Conclusion

Garlic contains bioactive substances that have substantial anticarcinogenic effects *via* a number of pathways, including cell cycle arrest, apoptosis, and stimulation of the angiogenic cascade. The nutritional and therapeutic benefits of garlic have been well-known. Among the numerous phytoconstituents of garlic, certain compounds, including SAC, allicin, DAS, SAMC, DATS, and DADS have strong anti-cancer activity. Derivatives of garlic and their nanoformulations resulted in altered expression levels of numerous re-ported genes associated with the regulation of several important signaling pathways, including the JNK, Akt/PI3K, p38, MAPK, EMT, Wnt, p53, ERK1/2, NF-κB, Nrf2, STAT3 and Chk1/cyclin B1 in human breast carcinoma. In a variety of preclinical cancer models, bioactive constituents of garlic and their nanoformulations decreased the cell proliferation, adhesion, apoptosis evasion, migration, invasion, and metastasis of several cancer types. While several signaling pathways have been targeted by various researchers for investigating the mechanism behind the anticancer efficacy of garlic phytocomponents, additional research is still required to fully comprehend these molecular targets of garlic and their derivatives inside diverse organ systems. Paucity of well-studied, potent clinical studies and thorough safety assessment, despite promising preclinical research on the anticancer potential of garlic components, increases the desire for additional clinical research. The nanoformulations of garlic demonstrated some efficient delivery methods with enhanced bioavailability. The efficacy of nanoparticles formulations of garlic constituents in the treatment of breast cancer was higher than compounds alone. However, more investigations are still needed to decipher the therapeutic potential of garlic components *via* utilizing crucial targets of cell signaling pathways for better management of human breast cancer in addition to well-controlled clinical investigations. Altogether, in this review, we have demonstrated the anticancer potential of garlic phytoconstituents and its nanoformulations as beneficial nutraceuticals and pharmaceuticals for the efficient management of human breast cancer.
