# Supplementary figures and images for: Updates on the anticancer potential of garlic organosulfur compounds and their nanoformulations: Plant therapeutics in cancer management
Source: Front Pharmacol. 2023 Mar 20;14:1154034. doi: 10.3389/fphar.2023.1154034 (PMC10067574; doi:10.3389/fphar.2023.1154034)

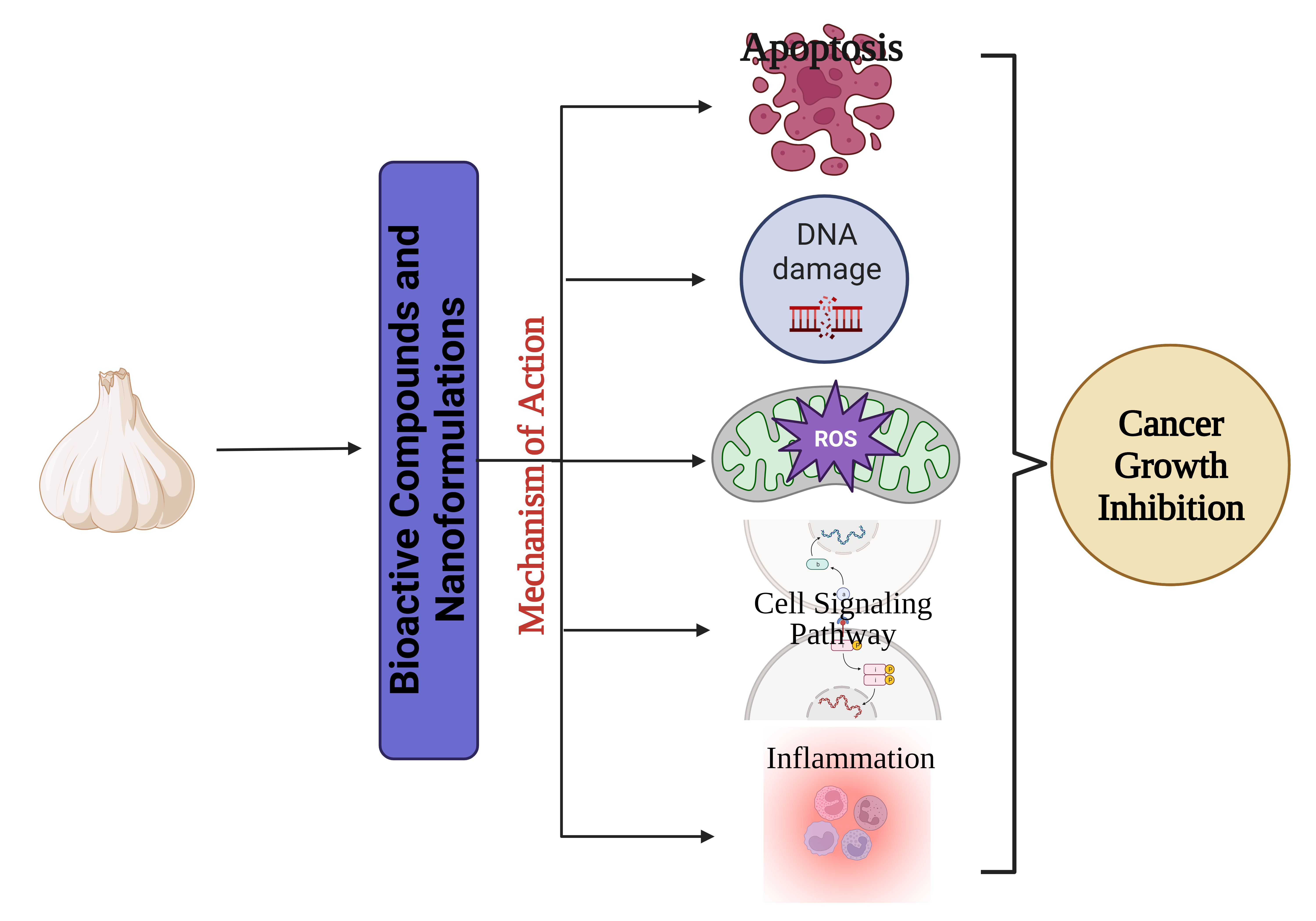

Supplement: Supplementary file 1 [file Image1.JPEG]
